# A Novel Fuzzy-Adaptive Extended Kalman Filter for Real-Time Attitude Estimation of Mobile Robots

**DOI:** 10.3390/s20030803

**Published:** 2020-02-01

**Authors:** Ákos Odry, Istvan Kecskes, Peter Sarcevic, Zoltan Vizvari, Attila Toth, Péter Odry

**Affiliations:** 1 Department of Control Engineering and Information Technology, University of Dunaújváros, Táncsics Mihály u. 1, 2400 Dunaújváros, Hungary; kecskes.istvan@gmail.com (I.K.); podry@uniduna.hu (P.O.); 2 Technical Department, Faculty of Engineering, University of Szeged, Moszkvai krt. 9, 6725 Szeged, Hungary; sarcevic@mk.u-szeged.hu; 3 Department of Environmental Engineering, Faculty of Engineering and Information Technology, University of Pécs, Boszorkány út 2, 7624 Pécs, Hungary; vizvari.zoltan@mik.pte.hu; 4 Institute of Physiology, Medical School, University of Pécs, Szigeti út 12, 7624 Pécs, Hungary; attila.toth@aok.pte.hu

**Keywords:** adaptive filter, attitude estimation, fuzzy logic, inertial measurement unit, extended Kalman filter, sensor fusion

## Abstract

This paper proposes a novel fuzzy-adaptive extended Kalman filter (FAEKF) for the real-time attitude estimation of agile mobile platforms equipped with magnetic, angular rate, and gravity (MARG) sensor arrays. The filter structure employs both a quaternion-based EKF and an adaptive extension, in which novel measurement methods are used to calculate the magnitudes of system vibrations, external accelerations, and magnetic distortions. These magnitudes, as external disturbances, are incorporated into a sophisticated fuzzy inference machine, which executes fuzzy IF-THEN rules-based adaption laws to consistently modify the noise covariance matrices of the filter, thereby providing accurate and robust attitude results. A six-degrees of freedom (6 DOF) test bench is designed for filter performance evaluation, which executes various dynamic behaviors and enables measurement of the true attitude angles (ground truth) along with the raw MARG sensor data. The tuning of filter parameters is performed with numerical optimization based on the collected measurements from the test environment. A comprehensive analysis highlights that the proposed adaptive strategy significantly improves the attitude estimation quality. Moreover, the filter structure successfully rejects the effects of both slow and fast external perturbations. The FAEKF can be applied to any mobile system in which attitude estimation is necessary for localization and external disturbances greatly influence the filter accuracy.

## 1. Introduction

### 1.1. Survey on Attitude Estimation

The microelectromechanical systems-based (MEMS-based) relative localization problem is a recent topic, which has been widely investigated in many areas including robotics and control [1,2,3,4,5,6,7,8], healthcare and rehabilitation [9,10,11], consumer electronics mobile devices [12,13,14], and automated driving and navigation [15,16,17,18], both in industry and in scientific research. Independent from the application, accurate and robust attitude estimation is a crucial task to be solved, especially if the results are to be incorporated into unstable closed-loop systems, such as the control algorithms of mobile robots and unmanned aerial vehicles (UAVs) [1].

The MEMS inertial measurement unit (IMU), composed of tri-axis MEMS accelerometer, gyroscope, and magnetometer sensors, also known as the measurement system of magnetic, angular rate, and gravity (MARG) sensor arrays, is the most commonly utilized device to track the real-time orientation of mobile platforms at present. The low-cost, low power consumption, and small size characteristics meet technological requirements, and therefore these devices have been widely utilized in embedded systems, where the filtering algorithm is executed by a microprocessor. As a result, an attitude and heading reference system (AHRS) has been formed, which provides the complete orientation measurement relative to the Earth’s gravitational and magnetic fields (global reference system), where the attitude denotes the roll and pitch angles, whereas heading refers to the yaw Euler angle [19]. The role of the aforementioned filtering algorithm is to combine the individual features of each sensor and provide both properly smoothed and robust attitude results with regard to the global reference system, in either Euler angles or quaternions. The most common method applied in sensor fusion techniques synthesizes the short-term accuracy of gyroscope-based attitude realizations and the accelerometer and magnetometer provide rough, low-frequency attitude corrections. This technique cancels the accumulated error (drift), smooths the signals, and produces long-term stable outputs if the IMU is in stationary states. Significant decrease in estimation performance arises when external disturbances are present, such as external accelerations, vibrations, and magnetic distortions, which prevent the utilization of the pure gravity and local magnetic field vectors in the calculation of the direction cosine matrix (DCM). The following paragraphs discuss the solutions provided in the literature.

Among recent developments, the Kalman filter (KF)—by different variants, such as stochastic approaches—and complementary filter (by frequency domain methods), both augmented with the intelligent use of deterministic techniques, have become the most popular methods for robust attitude determination [20]. Deterministic techniques have been shown to solve Wahba’s problem [21] and provide attitude estimation based on gravity and magnetic field observations. The fundamental solutions are three-axis attitude determination (TRIAD), which produces suboptimal attitude matrix estimation by the construction of two triads of orthonormal unit vectors, and the QUaternion ESTimator (QUEST), in which the quaternion is found by minimizing a quadratic gain function based on a set of reference and observation vectors. Improved approaches have utilized the fast optimal matrix algorithm (FOAM) [22], the factored quaternion algorithm (FQA) [23], the Gauss–Newton algorithm [24], Levenberg Marquardt algorithm [25], the gradient descent algorithm [26], and the super fast least-squares optimization-based algorithm [27]. Each approach estimates the attitude based on accelerometer and magnetometer measurements and is characterized by reduced computational complexity or more robust performance. As the estimation performance significantly decreases with disturbances (magnetic perturbation and/or external acceleration), the incorporation of gyroscope measurements has thus become a de facto standard for the state propagation.

Complementary filters (CF) use frequency domain information to synthesize signals that have complementary spectral components. This concept enables us to combine the slowly varying signals of the accelerometer and magnetometer with the fast signals of the gyroscope through low- and high-pass filters, respectively. The CF has been widely implemented in the robotics and control community, due to its simple structure and ease of implementation [28,29]. In [28], a nonlinear CF was developed for UAVs, which also employed first-order vehicle dynamics to cancel the effect of external acceleration. A quaternion-based nonlinear CF (qNCF) for attitude estimation was developed in [30] (hereafter referred to as the Mahony filter), which corrects the gyroscope measurements with a proportional and integral (PI) controller and provides attitude and gyroscope bias estimates. The popular Madgwick filter [26] is a computationally efficient constant gain filter, which was developed originally for human motion tracking applications. The filter has been improved recently in [7], employing the accelerometer and magnetometer measurements in a gradient descent algorithm to correct the quaternion obtained through the integration of rate measurements. Mahony and Madgwick filters are widely utilized algorithms and their performances have regularly been considered in comparative analyses [9,13,15,31,32,33]. In [34], an adaptive-gain CF was proposed to provide good estimates, even in dynamic or high-frequency situations. The filter gain was modified based on both the convergence and divergence rates of observation-based orientation realization and gyroscope-based orientation propagation, respectively. An improved qCF was designed in [32], in which two correction sequences were employed based on separating the quaternion into accelerometer- and magnetometer-based realizations. Moreover, the algorithm was augmented with an adaptive gain characterized by two thresholds to reduce the estimation error when dynamic motion is present. The filter performance was validated with experiments containing short external disturbances. This algorithm was adapted in [10], where its real-time performance was evaluated on a microprocessor-controlled lower limb prosthesis. An iteration-free variant of CF has been proposed for efficient attitude estimation calculation in [35], where a linear system was employed for the accelerometer-based attitude realization. The filter performance was evaluated under different conditions and the effects of vibration and magnetic distortion were examined as well. However, the developed CF was not as accurate as the benchmark KF, especially under highly dynamic conditions. In [36], a two-step qCF was implemented for human motion tracking applications. The algorithm was characterized by two separate tuning parameters; moreover, it contained a finite state machine-based adaptive strategy to cope with external disturbances. The two-step configuration made the attitude output more resistant to magnetometer measurements, as the attitude was obtained based on accelerometer and gyroscope data first, following which the heading angle was updated using both the estimate and magnetometer data.

The KF and its extension for nonlinear cases, the extended KF (EKF), are the most prevalent Bayesian state estimation algorithms utilized for attitude determination. These recursive algorithms deal with statistical descriptions and predict the state of the Gaussian stochastic model of MARG with minimum variance. The main performance, which includes both the filter dynamics and convergence, is determined with the proper covariance matrices that describe the stochastic system. In [37], a qEKF was developed for human movement tracking, in which the state of a rotation quaternion was augmented with the random walk processes of accelerometer and magnetometer bias vectors. Moreover, an adaptive strategy modified the noise covariance matrix if an external disturbance was identified. The filter was improved by modeling the magnetic variations with a Gauss–Markov vector random process, which aimed to reduce the effect of fluctuating magnetic environments [38]. Adaptive threshold-based switching strategies have been used to modify the covariance matrices based on the measured stationary-, low-, and high-acceleration modes in [39,40]. In [19], an acceleration model was incorporated in the stochastic model, and thus the KF both estimated and compensated for the external acceleration in an attitude determination process. The proposed method was evaluated under dynamic conditions and compared with a threshold-based KF; however, significant improvement in the estimation accuracy was not highlighted. In [14,41], smartphone-based human body orientation estimation was addressed with the application of a qAEKF. The proposed adaptive strategy modified the noise covariance matrix based on the variance of input signal. Moreover, the upper and lower bounds of covariance values were selected by numerical optimization. Comparison with both the Android OS algorithm and a simple CF highlighted the benefits of the proposed method. A similar qEKF structure without adaptation laws was proposed for the attitude estimation of UAVs in [42]. The filter was set up with experimentally tuned noise covariance matrices; however, its performance was evaluated without external dynamic effects on a multi-function turntable device. A reduced state vector-based qEKF approach was applied in [3], in which the measurement noise covariance was tuned in real-time, based on the angle between the predicted and measured gravitational accelerations. A two-step geometrically-intuitive quaternion correction was proposed for a linear KF, which enabled isolation of the pitch and roll estimation performance from magnetic distortion effects by decoupling the accelerometer and magnetometer data [43]. In [44], a linear KF was implemented for human motion tracking applications in dynamic environments. In their real-world experiments, the effects of long external accelerations were addressed and good overall performance was achieved by the filter; however, significant error peaks were present in the estimation as well. A smart detector augmented AEKF was proposed in [45] with similar filter efficiency. The adaptive strategy identified both static and dynamic body motions. Moreover, the effect of external acceleration was suppressed through filter gain tuning. The attitude estimation problem during sports activities was addressed in [46], where the proposed EKF considered the model uncertainty of active acceleration. Experiments highlighted the robustness of the approach, especially when large accelerations were present during the tests. In [47], the maneuvering target tracking problem was addressed and the application of both General Regression Neural Networks (GRNN) and an additional maneuver detector algorithm was proposed for the state estimation of manoeuvring objects. Moreover, a comparison of the GRNN-based neural filter and KF for target movement vector estimation was presented in [48,49], where the GRNN-based approach was characterized by superior estimation performance only during steady motions. In [50], a fuzzy inference system was proposed to tune the noise covariance matrix of the EKF based on the filter innovation sequence through a covariance-matching technique. The experimental results showed that the fuzzy rule-based adaptive strategy effectively improved the estimation accuracy with respect to the standard EKF algorithm. In [51], an adaptive analytical algorithm was presented for the determination of UAV orientation angles. The algorithm employed both MARG and GPS-based correction channels; moreover, an UAV maneuver intensity classification method was implemented to increase the orientation estimation performance.

Recent studies have proposed the use of unscented KF (UKF) over EKF [52,53], and stated that UKF-based approaches better deal with the high-order nonlinear terms of large attitude errors. Attitude estimation has been solved with computationally efficient geometric UKF [53], where a new formulation of the UKF algorithm was proposed in [52] to maintain fast and slow variations in the measurement uncertainty. The latter algorithm was augmented with both an adaptive strategy to tune the covariance matrices on-the-fly and an outlier detector to reject the effects of external disturbances. An industrial manipulator robot was used to conduct the experiments, where the algorithm provided superior performance over the standard UKF and Madgwick filters. Recent developments have considered the MARG as a non-Gaussian stochastic system and developed maximum correntropy KF (MCKF) for attitude estimation [54,55]. These algorithms employed the MC criterion, instead of the minimum mean square error, to estimate the state of the system corrupted by non-Gaussian impulsive noises. However, the comprehensive case study provided in [56] has not highlighted the superior state estimation performance of the MCC-based techniques in non-Gaussian noise environments.

Based on the methods discussed above, it can be concluded that the ultimate attitude estimation quality is determined by three main factors:

1. The first impact is related to the flexibility of the implemented algorithm (i.e., the observation models, equations defining the filter dynamics, and noise models jointly define the algorithm).
2. The filter performance heavily depends on properly selected filter gains (i.e., noise covariance matrices). In general, the statistics of system noise cannot be determined; moreover, external disturbances cause radical measurement noise during attitude realization, which make the assumed noise models inappropriate. As a result, the filter parameters are usually selected based on both experimental and engineering intuition, which result in a compromise between the accuracy and filter dynamics. To enhance the filter performance, numerical optimization-based filter tuning has been performed [1,14,40,57].
3. The papers above show that the common methods used to deal with external disturbances (dynamic motions and magnetic perturbations) either work by the application of intelligent adaptive strategies that on-the-fly modify the vector observation methods, filter gains, and covariance matrices; or the compensation is maintained with additional dynamic models that well-mimic the effects of the external forces and magnetic fluctuations.

### 1.2. Contribution of the Paper

This paper addresses the robust attitude estimation problem for mechatronic systems (robots) characterized by fast dynamics, unstable equilibria, and/or mechanical difficulties (e.g., the driving mechanism backlash). For these type of systems, reliable state estimation is both an essential and crucial task to be solved, as the unstable dynamics are stabilized in closed-loop with a control algorithm, in which the stabilizing system inputs are calculated based on the estimation results. If the state estimation contains significant errors, then these control signals will drive the system out of equilibrium to unwanted states, which may eventually damage the system and its environment [1,42].

The aforementioned discussion highlights that providing both reliable and robust attitude estimates, especially for extreme dynamic situations, remains an important issue. For this problem, our paper proposes a novel qAEKF, in which new methods are employed to measure the external disturbances and their effect is suppressed with adaptation laws described with fuzzy logic-based IF-THEN rules. The results show that the proposed methods significantly improve the robustness of the state estimation, both in static and extremely vibrating and accelerating environments. Moreover, to the author’s best knowledge, no study has yet investigated the attitude estimation problem in such dynamic environments. The basis of the proposed filter structure was presented in [1], where the techniques were validated for one-dimensional attitude estimation using a linear KF. That investigation showed promising results, thereby motivating us to extend the estimation problem to the complete orientation based on MARG systems. The novelties of the paper are summarized as follows.

1. Formulating a novel quaternion-based FAEKF structure that incorporates the magnitudes of vibration, external acceleration, and magnetic perturbation by a sophisticated heuristic knowledge-based fuzzy inference machine to provide robust attitude estimation in both static and dynamic environments.
2. Designing a 6 DOF test platform which enables both the execution of various dynamic (vibrating and accelerating) behaviors in the three-dimensional space and the measurement of true attitude angles along with the raw MARG data. An additional part of the test environment is a novel magnetic perturbation algorithm. This test environment contributes to the successful evaluation of state estimation quality.
3. Performing numerical optimization-aided tuning of filter parameters based on the collected training measurements in the test environment.
4. Providing a free-to-use Robot Operating System (ROS) package that enables both the generation of MARG-based measurements and the testing of filter performances. We made this package publicly available on our website [58], with the aim of helping other laboratory teams with both performing and developing similar experiments.

The proposed approaches can be advantageously applied in such mechatronic systems where accurate attitude determination is crucial for the closed-loop dynamics; moreover, where external disturbances are frequently present, due to fast maneuvers, collision, or unstable dynamics.

The remainder of the paper is organized as follows. Section 2 gives an introduction to quaternion representation and highlights the important relationships. In Section 3, the stochastic models of MARG sensor arrays are discussed and a suitable EKF formulation for attitude estimation is described. Section 4 presents the fuzzy adaptive strategy in detail, in which external disturbance magnitudes are measured with three novel methods; additionally, a sophisticated fuzzy inference machine is employed to manipulate the noise variances consistently. Section 5 introduces the test bench which was designed for estimation quality evaluation, the optimization-aided tuning of filter parameters, and the experimental results of the proposed approaches. Finally, in Section 6, the conclusions and recommendations for future studies are discussed.

## 2. Quaternion-Based Attitude Formulation

**Figure 1:**
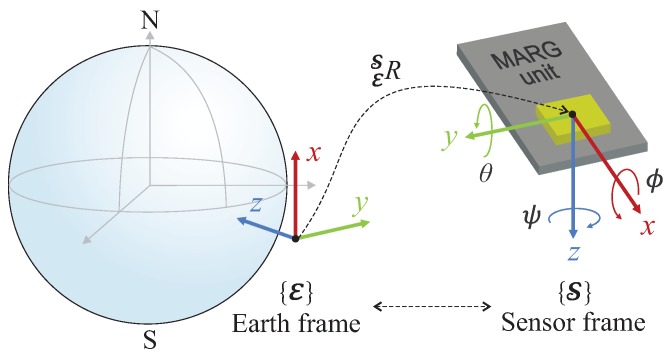
Relative orientation between the earth frame (E) and sensor frame (S).

Let E and S denote the earth and sensor frames, also called the global non-moving inertial and local mobile frames, respectively. These frames can be defined with the conventional North-East-Down (NED) configuration often applied for robotic applications [3,5,42]. Namely, the *x*-axis points north and *y* is directed east, whereas *z* completes the right-handed coordinate system by pointing down in the inertial reference frame (see Figure 1). Additionally, the origin of the right-handed sensor frame is attached to the center of mass of the moving body, where the *x*-axis points forward and the *y*-axis is directed to the right of the body. The mapping between these frames E and S is described by a rotation matrix as

(1) $${}_{}^{E}x{=}_{S}^{E}R_{}^{S}x,$$

where ${}_{}^{E}x$ and ${}_{}^{S}x$ denote the 3×1 vector observations in the earth and sensor frames, respectively. Moreover, ${}_{S}^{E}R\in \mathrm{SO}\left(3\right)$ indicates the 3×3 special orthogonal matrix, where the inverse transformation is defined as ${}_{S}^{E}R^{-1}{=}_{S}^{E}R^{T}{=}_{E}^{S}R$.

A quaternion representation provides an effective way to both formulate the aforementioned rotation matrix and describe the attitude of the coordinate frames in three-dimensional space [59]. The advantageous structure both provides fast computation (compared to DCM) and completely avoids the well-known singularity problem of Euler angles (also known as the gimbal lock problem) [60]. The unit quaternion formulated by the four-dimensional vector ${}_{S}^{E}q\in R^{4}$, $∥{}_{S}^{E}q∥=1$ describes the attitude of frame E relative to frame S as a rotation by an angle μ about the unit vector $e={\left(e_{x},e_{y},e_{z}\right)}^{T}$, which represents the rotation axis in S. This rotation quaternion is interpreted as ${}_{S}^{E}q={\left(\cos\frac{\mu }{2},e^{T}\;\cdot \;\sin\frac{\mu }{2}\right)}^{T}={\left(q_{0},\varrho \right)}^{T}$, where $q_{0}$ and $\varrho ={\left(q_{1},q_{2},q_{3}\right)}^{T}$ denote the scalar and vector part terms, respectively. Co-ordinate transformation is performed by the non-commutative quaternion product denoted by ⊗:

(2) $${}_{}^{E}x{=}_{S}^{E}q{⊗}_{}^{S}x{⊗}_{S}^{E}q^{*}.$$

In Equation (2), ${}_{S}^{E}q^{*}={\left(q_{0},-\varrho \right)}^{T}$ denotes the conjugate quaternion that describes the attitude of frame S relative to frame E (i.e., the inverse rotation is formulated as ${}_{S}^{E}q^{*}{=}_{E}^{S}q$). Moreover, ${}_{}^{E}x$ and ${}_{}^{S}x$ indicate the quaternions associated with the vector observations by their augmentation with zero scalar parts ($q_{0}=0$) as $x={\left(0,x^{T}\right)}^{T}$. The rotation can be rearranged into the initial Equation (1) with the quaternion-parameterized rotation matrix

(3) $$\begin{array}{cc} {}_{S}^{E}R\left(q\right) & =\left(q_{0}^{2}-\varrho^{T}\varrho \right)I_{3}+2\varrho \varrho^{T}+2q_{0}\left[\varrho \times \right]\\ \; & =\left[\begin{array}{ccc} q_{0}^{2}+q_{1}^{2}-q_{2}^{2}-q_{3}^{2} & 2\left(q_{1}q_{2}-q_{0}q_{3}\right) & 2\left(q_{1}q_{3}+q_{0}q_{2}\right)\\ 2\left(q_{1}q_{2}+q_{0}q_{3}\right) & q_{0}^{2}-q_{1}^{2}+q_{2}^{2}-q_{3}^{2} & 2\left(q_{2}q_{3}-q_{0}q_{1}\right)\\ 2\left(q_{1}q_{3}-q_{0}q_{2}\right) & 2\left(q_{2}q_{3}+q_{0}q_{1}\right) & q_{0}^{2}-q_{1}^{2}-q_{2}^{2}+q_{3}^{2} \end{array}\right], \end{array}$$

where $I_{3}$ is the identity matrix of size 3 and [ϱ×] denotes the antisymmetric matrix of ϱ, defined for the vector cross product ϱ×x=[ϱ×]x as

(4) $$\left[\varrho \times \right]=\left[\begin{array}{ccc} 0 & -q_{3} & q_{2}\\ q_{3} & 0 & -q_{1}\\ -q_{2} & q_{1} & 0 \end{array}\right].$$

Let ${}_{}^{S}\omega ={\left(0,\omega_{x},\omega_{y},\omega_{z}\right)}^{T}$ denote the four-dimensional quaternion formed by the angular velocities about the *x*, *y*, and *z* axes in the sensor frame. The time derivative of the quaternion ${}_{S}^{E}q$ represents the rate of change of attitude E relative to frame S, according to the vector differential equation

(5) $${}_{S}^{E}\dot{q}={\frac{1}{2}}_{S}^{E}q{⊗}_{}^{S}\omega =\frac{1}{2}Q{\left(q\right)}_{}^{S}\omega ,\;Q\left(q\right)=\left[\begin{array}{cc} q_{0} & -\varrho^{T}\\ \varrho & q_{0}I_{3}+\left[\varrho \times \right] \end{array}\right],$$

where the matrix-vector product is indicated by the quaternion matrix $Q\left(q\right)$. The attitude of frame E relative to S is obtained by integrating the quaternion derivative ${}_{S}^{E}\dot{q}$. Thereforeforth, the sub- and super-scripts are omitted, for the sake of simplicity.

The authors used the Euler angles for the quality evaluation of attitude estimation, as their interpretation is straightforward for the reader. Euler angles (including yaw, pitch, and roll) describe the attitude as a sequence of three rotations, where ψ, θ, and ϕ denote the rotation angles about the *z*, *y*, and *x* axes, respectively. The quaternion output provided by the analyzed filters was converted to Euler representation as follows.

(6) $$\begin{array}{cc} \phi & =arctan2\left(2q_{2}q_{3}-2q_{0}q_{1},2q_{0}^{2}+2q_{3}^{2}-1\right),\\ \theta & =-\tan^{-1}\left(\frac{2q_{0}q_{2}+2q_{1}q_{3}}{\sqrt{1-{\left(2q_{0}q_{2}+2q_{1}q_{3}\right)}^{2}}}\right),\\ \psi & =arctan2\left(2q_{1}q_{2}-2q_{0}q_{3},2q_{0}^{2}+2q_{1}^{2}-1\right). \end{array}$$

## 3. Attitude Estimation with MEMS MARG Sensors

Each sensor of a MEMS-based MARG unit provides useful information of the instantaneous attitude; however, none of the sensors are capable of providing reliable attitude results alone. Gyroscopes measure angular velocities; therefore, gyroscope-based attitude realization is obtained through numerical integration, but both the temperature-dependent bias and noise contained in the measurements cause cumulative errors. An accelerometer measures the sum of gravitational and external accelerations. In stationary states, long-term stable attitude realization can be obtained based on the decomposition of the sensed gravity vector but, as external accelerations increase as a result of dynamic motion, the quality of attitude realization drastically deteriorates, making accelerometer-based realization highly unreliable. Magnetometers measure the geomagnetic field, which is used to determine heading information. However, the magnetic fluctuation of the environment caused by the perturbation of ferromagnetic objects highly disturbs the magnetometer output.

To provide reliable attitude estimation results, the individual features of each sensor are carefully addressed in the following.

### 3.1. Gyroscope Model

Let $\Omega_{k}$ denote the raw measurement vector of a tri-axis MEMS gyroscope in the $k^{\mathrm{th}}$ time instance. This measurement vector is composed of a 3×1 vector $\omega_{k}$ of true angular velocities around the *x*, *y*, and *z* axes, a vector ${\bar{\omega }}_{k}$ containing the non-static bias terms, and a vector $\mu_{k}$ of additive measurement noises. The imperfections of manufacturing results, in that the sensor model is extended with axis misalignment and scale factor errors, are represented by the 3×3 matrices $M_{\Omega }$ and $\Delta S_{\Omega }$, respectively. Moreover, the temperature sensitivity of the sensor makes the slowly varying bias vector ${\bar{\omega }}_{k}$ propagate as a random walk process characterized by a driving noise vector $\eta_{k}$, and therefore [61]

(7) $$\begin{array}{cc} \Omega_{k} & =\left(I+\Delta S_{\Omega }\right)M_{\Omega }\omega_{k}+{\bar{\omega }}_{k}+\mu_{k},\\ {\bar{\omega }}_{k} & ={\bar{\omega }}_{k-1}+\eta_{k}. \end{array}$$

in the above measurement model, the rate noise vectors contain zero-mean white Gaussian variables for each axis (i.e., $E\left[\mu_{k}\right]=E\left[\eta_{k}\right]=0$) and the covariance matrices are defined as $E\left[\mu_{k}\mu_{l}^{T}\right]=\Sigma_{\mu ,k}\delta_{kl}$, $\Sigma_{\mu ,k}\geq 0$, and $E\left[\eta_{k}\eta_{l}^{T}\right]=\Sigma_{\eta ,k}\delta_{kl}$, $\Sigma_{\eta ,k}\geq 0$, where $\delta_{kl}$ denotes the Kronecker delta.

Gyroscope-based (gyro-based) attitude realization is obtained by numerical integration of the true angular velocity vector $\omega_{k}$ in Equation (7). Common calibration procedures performed in laboratories allow for the determination and compensation of the scale factor and misalignment errors. This process exceeds the scope of this article; therefore, we assume that the compensation has already been performed ($M_{\Omega }=I$ and $\Delta S_{\Omega }=0$) [22,41,62]. Based on Equation (5), the gyro-based attitude realization is given in quaternion form as

(8) $$q_{k+1}=q_{k}+\frac{T_{s}}{2}Q\left(q_{k}\right)\left[\begin{array}{c} 0\\ \Omega_{k}-{\bar{\omega }}_{k} \end{array}\right],$$

where $T_{s}=1/f_{s}$ is the sampling time. However, this method yields only short-tem accuracy, due to the presence of bias and measurement noise terms (${\bar{\omega }}_{k}$ and $\mu_{k}$) resulting in boundless drift in the attitude propagation.

### 3.2. Accelerometer and Magnetometer Models

The accelerometer and magnetometer sensors provide absolute reference observations, and therefore their measurements can be combined to determine the complete attitude of the sensor. The raw output $A_{k}$ of a tri-axis MEMS accelerometer consists of four main components: the gravitational and external acceleration vectors $g_{k}$ and $\alpha_{k}$ measured in the sensor frame (S), the vector $a_{0}$ of bias terms, and the vector $\nu_{k}$ of additive measurement noises. Additionally, the raw measurement vector $H_{k}$ of the tri-axis MEMS magnetometer model is composed of the true local magnetic field $h_{k}$ sensed in S, the sensor bias vector $h_{0}$, and the measurement noise vector $\epsilon_{k}$:

(9) $$\begin{array}{cc} A_{k} & =\left(I+\Delta S_{A}\right)M_{A}\left(\alpha_{k}+g_{k}\right)+a_{0}+\nu_{k},\\ H_{k} & =\left(I+\Delta S_{H}\right)M_{H}\left(B_{si}h_{k}+b_{hi}\right)+h_{0}+\epsilon_{k}. \end{array}$$

Similarly to the gyroscope model, Gaussian noises are considered in the aforementioned models; therefore, $E\left[\nu_{k}\right]=E\left[\epsilon_{k}\right]=0$ and the covariance matrices are $E\left[\nu_{k}\nu_{l}^{T}\right]=\Sigma_{\nu ,k}\delta_{kl}$, $\Sigma_{\nu ,k}\geq 0$ and $E\left[\epsilon_{k}\epsilon_{l}^{T}\right]=\Sigma_{\epsilon ,k}\delta_{kl}$, $\Sigma_{\epsilon ,k}\geq 0$. Beside the scaling and misalignment errors ($\Delta S_{A}$, $\Delta S_{H}$, $M_{A}$, and $M_{H}$), the magnetometer measurements are disturbed by magnetic soft iron and hard iron errors caused by the local environment, represented by the 3×3 matrix $B_{si}$ and the 3×1 vector $b_{h}$, respectively. These model errors are determined via self-calibration procedures which address the time-invariant nature of the vector fields and map the distribution of the measurements on an ellipsoid [63,64,65]. We assume that the compensation has already been performed (therefore, $h_{k}:=B_{si}^{-1}h_{k}-b_{hi}$), the bias and scale errors are zero, and the misalignment errors are identity matrices.

If a mobile mechatronic system stays in stationary states (i.e., no external acceleration is performed; $\alpha_{k}\approx 0$) and, moreover, if the local magnetic field is not perturbed by ferromagnetic objects, then the locally constant reference vectors can express the observations, with the help of the rotation matrix, as

(10) $$\begin{array}{cc} {}_{}^{S}A_{k} & {=}_{E}^{S}{R\left(q_{k}\right)}_{}^{E}g,\\ {}_{}^{S}H_{k} & {=}_{E}^{S}{R\left(q_{k}\right)}_{}^{E}h. \end{array}$$

In the aforementioned configuration, the gravity vector is given as ${}_{}^{E}g={\left(0,0,9.81\right)}^{T}$, whereas the magnetic field vector is ${}_{}^{E}h={\left(b\cos\left(\sigma \right),0,b\sin\left(\sigma \right)\right)}^{T}$ in SI units, where *b* and σ denote the magnitude of the Earth’s geomagnetic field and inclination angle, respectively.

Let the components of an inertial frame in both S and E be expressed by constructing two triads of orthonormal unit vectors. The first triad is defined with the reference vectors in E as

(11) $$\begin{array}{c} {\hat{s}}_{1}=\frac{{}_{}^{E}g}{∥{}_{}^{E}g∥},\;{\hat{s}}_{2}=\frac{{}_{}^{E}g\times_{}^{E}h}{∥{}_{}^{E}g\times_{}^{E}h∥},\;{\hat{s}}_{3}={\hat{s}}_{1}\times {\hat{s}}_{2}. \end{array}$$

The second triad is constructed with the observation vectors in frame S, where

(12) $$\begin{array}{c} {\hat{r}}_{1}=\frac{{}_{}^{S}A_{k}}{∥{}_{}^{S}A_{k}∥},\;{\hat{r}}_{2}=\frac{{}_{}^{S}A_{k}\times_{}^{S}H_{k}}{∥{}_{}^{S}A_{k}\times_{}^{S}H_{k}∥},\;{\hat{r}}_{3}={\hat{r}}_{1}\times {\hat{r}}_{2}. \end{array}$$

Based on Equations (10)–(12), first the measurement (observation) and reference matrices are formed, then the rotation matrix is determined as:

(13) $$\begin{array}{c} M_{\mathrm{mea}}=\left[{\hat{r}}_{1}\;{\hat{r}}_{2}\;{\hat{r}}_{3}\right],\;M_{\mathrm{ref}}=\left[{\hat{s}}_{1}\;{\hat{s}}_{2}\;{\hat{s}}_{3}\right],{\;}_{E}^{S}R\left(q_{k}\right)=M_{\mathrm{mea}}M_{\mathrm{ref}}^{T}. \end{array}$$

The determined rotation matrix ${}_{E}^{S}R\left(q_{k}\right)=\left(r_{ij}\right)$ enables the calculation of the quaternion representing the attitude of the sensor frame:

(14) $$\begin{array}{c} q_{0}=\frac{1}{2}\sqrt{1+r_{11}+r_{22}+r_{33}},\;q_{1}=\frac{r_{23}-r_{32}}{4q_{0}},\;q_{2}=\frac{r_{31}-r_{13}}{4q_{0}},\;q_{3}=\frac{r_{12}-r_{21}}{4q_{0}}. \end{array}$$

The aforementioned algorithm is the well-known TRIAD [22,66], which produces the raw attitude realization based on accelerometer and magnetometer measurements. The attitude realization, which is described by Equation (14), is denoted by $q_{k,\mathrm{TRIAD}}={\left(q_{0},q_{1},q_{2},q_{3}\right)}^{T}$ and can also be considered as the sum of the real attitude characterized by the quaternion $q_{k}$ in the $k^{\mathrm{th}}$ time instance and an additive Gaussian white noise, $v_{k}$, which represents the effects of $\nu_{k}$ and $\epsilon_{k}$ from Equation (9) after the TRIAD output is evaluated:

(15) $$\begin{array}{c} q_{k,\mathrm{TRIAD}}=q_{k}+v_{k},\;E\left[v_{k}\right]=0,\;E\left[v_{k}v_{l}^{T}\right]=\Sigma_{v,k}\delta_{kl},\Sigma_{v,k}>0. \end{array}$$

This algorithm is characterized by a simple and straightforward implementation and, therefore, it is a popular choice for raw attitude determination [2,3]. However, it has a disadvantage in producing large errors when dynamic conditions are present or external magnetism disturbs the sensor readings. As a result, if external acceleration is performed ($\alpha_{k}\neq 0\to_{}^{S}A\neq R_{}^{E}g$) or ferromagnetic materials distort the geomagnetic field (${}_{}^{S}H\neq R_{}^{E}h$), then the attitude realization becomes unreliable with drastically reduced accuracy. This implementation method does not include any explicit models of external disturbances. Instead, the effects of external disturbances are absorbed by $v_{k}$ in Equation (15); that is, the additive noise is characterized by a significantly larger noise variance in disturbed environments.

### 3.3. Attitude Estimation with Extended Kalman Filter

The MARG sensor-based attitude realizations described by Equations (8) and (15) are utilized in a sensor fusion algorithm, which both synthesize the individual advantages and features of each sensor and provides attitude results with higher reliability and accuracy. First, this sensor fusion algorithm utilizes the gyroscope-based realization to propagate the attitude results, then these results are updated with the most recent quaternion realization derived from accelerometer and magnetometer readings. This propagate-update mechanism provides both a smooth output and stability in the attitude results by compensating for the drift error generated in Equation (8). The fusion of the sensor models is executed with an EKF.

The EKF effectively combines the noisy measurements and dynamic model-based predictions; moreover, in a recursive filter structure, it provides an approximate maximum-likelihood state estimate $\hat{x}$ of the stochastic nonlinear state-space model [22]. In fact, the filter linearizes the nonlinear dynamic model around the last estimated state vector using the Jacobian matrix and, for the linearized dynamics, the linear KF is utilized, which is an the optimal state estimator such that $E\left[x_{k}-{\hat{x}}_{k}\right]=0$ and $E\left[\left(x_{k}-{\hat{x}}_{k}\right){\left(x_{k}-{\hat{x}}_{k}\right)}^{T}\right]\to \inf$.

The mathematical models and statistical assumptions of MARG sensors, as introduced in the previous subsections, fully match the process and measurement equations of a stochastic nonlinear state-space model. Namely, the process model describes the quaternion propagation with both the discrete-time integrated angular velocities (Equation (8)) and the random walk process of the bias term (Equation (7)). Therefore, the dynamic model is defined with the 7×1 state vector $x_{k}={\left(q_{k},{\bar{\omega }}_{k}\right)}^{T}$, the 3×1 input vector $u_{k}=\Omega_{k}$, and the 7×1 process noise vector $w_{k}={\left(\mu_{k}^{q},\eta_{k}\right)}^{T}$, where $\mu_{k}^{q}$ represents the quaternion noise generated due to the gyroscope measurement noise $\mu_{k}$. For the sake of comprehensiveness and to foster a straightforward implementation, we give the full description of state propagation in Equation (16):

(16) $$\begin{array}{cc} x_{k+1} & =f\left(x_{k},u_{k},w_{k}\right),\;x\left(0\right)\\ {\left[\begin{array}{c} q_{0}\\ q_{1}\\ q_{2}\\ q_{3}\\ {\bar{\omega }}_{x}\\ {\bar{\omega }}_{y}\\ {\bar{\omega }}_{z} \end{array}\right]}_{k+1}\; & =\left[\begin{array}{c} q_{0,k}+\frac{T_{s}}{2}\left(q_{1,k}\left({\bar{\omega }}_{x,k}-\Omega_{x,k}\right)+q_{2,k}\left({\bar{\omega }}_{y,k}-\Omega_{y,k}\right)+q_{3,k}\left({\bar{\omega }}_{z,k}-\Omega_{z,k}\right)\right)+\mu_{0,k}^{q}\\ q_{1,k}-\frac{T_{s}}{2}\left(q_{0,k}\left({\bar{\omega }}_{x,k}-\Omega_{x,k}\right)-q_{3,k}\left({\bar{\omega }}_{y,k}-\Omega_{y,k}\right)+q_{2,k}\left({\bar{\omega }}_{z,k}-\Omega_{z,k}\right)\right)+\mu_{1,k}^{q}\\ q_{2,k}-\frac{T_{s}}{2}\left(q_{3,k}\left({\bar{\omega }}_{x,k}-\Omega_{x,k}\right)+q_{0,k}\left({\bar{\omega }}_{y,k}-\Omega_{y,k}\right)-q_{1,k}\left({\bar{\omega }}_{z,k}-\Omega_{z,k}\right)\right)+\mu_{2,k}^{q}\\ q_{3,k}+\frac{T_{s}}{2}\left(q_{2,k}\left({\bar{\omega }}_{x,k}-\Omega_{x,k}\right)-q_{1,k}\left({\bar{\omega }}_{y,k}-\Omega_{y,k}\right)-q_{0,k}\left({\bar{\omega }}_{z,k}-\Omega_{z,k}\right)\right)+\mu_{3,k}^{q}\\ {\bar{\omega }}_{x,k}+\eta_{x,k}\\ {\bar{\omega }}_{y,k}+\eta_{y,k}\\ {\bar{\omega }}_{z,k}+\eta_{z,k} \end{array}\right] \end{array}$$

According to Equation (15), the measurement model is characterized by a linear quaternion mapping. Therefore, it is formed with the 4×1 output vector $z_{k}=q_{k,\mathrm{TRIAD}}$ which provides the quaternion update as the TRIAD output, the measurement noise vector $v_{k}$, and the output matrix *H*, as

(17) $$\begin{array}{cc} z_{k} & =Hx_{k}+v_{k},\\ q_{k,\mathrm{TRIAD}} & =\left[\begin{array}{cc} I_{4} & 0_{4\times 3} \end{array}\right]\left[\begin{array}{c} q_{k}\\ {\bar{\omega }}_{k} \end{array}\right]+v_{k}. \end{array}$$

If the $x\left(0\right)$ Gaussian vector in Equation (16) is known along with its mean and covariance matrix; that is, if

(18) $$\begin{array}{c} {\hat{x}}_{0}=E\left[x\left(0\right)\right],\;P_{0}=E\left[\left(x\left(0\right)-{\hat{x}}_{0}\right){\left(x\left(0\right)-{\hat{x}}_{0}\right)}^{T}\right], \end{array}$$

then the MARG sensor models fully satisfy the stochastic hypothesis. Namely, the process and measurement noise vectors are zero-mean white Gaussian variables, $x\left(0\right)$ is uncorrelated to $w_{k}$ and $v_{k}$, and, moreover,

(19) $$\begin{array}{c} E\left[w_{k}v_{l}^{T}\right]=0,\;E\left[w_{k}w_{l}^{T}\right]=Q\delta_{kl},\;E\left[v_{k}v_{l}^{T}\right]=R\delta_{kl}, \end{array}$$

where Q≥0 and R>0 are the process and measurement noise covariance matrices, respectively. The EKF algorithm provides a suboptimal state estimation ${\hat{x}}_{k}$ with minimized estimation error covariance. The state propagation, processing of the observations, and the covariance estimate update are performed through time and measurement update equations in the recursive filter structure; namely, the time update equations utilize the input variable $u_{k}$, the state estimation and error covariance obtained in the previous step (${\hat{x}}_{k-1}$ and $P_{k-1}$), and the state dynamics $f\left({\hat{x}}_{k-1},u_{k}\right)$ to calculate the a priori state estimate (${\hat{x}}_{k}^{-}$) and the corresponding error covariance ($P_{k}^{-}$):

(20) $$\begin{array}{cc} {\hat{x}}_{k}^{-} & =f\left({\hat{x}}_{k-1},u_{k}\right),\\ P_{k}^{-} & =\Phi P_{k-1}\Phi^{T}+Q,\;\Phi =\frac{\partial f}{\partial x}{|}_{{\hat{x}}_{k-1}}. \end{array}$$

in Equation (20), the Jacobian Φ is applied in the a priori covariance matrix update. To foster straightforward implementation, we give its full form as follows,

(21) $$\begin{array}{c} \Phi =\left[\begin{array}{ccccccc} 1 & \frac{T_{s}}{2}\left({\bar{\omega }}_{x}-\Omega_{x}\right) & \frac{T_{s}}{2}\left({\bar{\omega }}_{y}-\Omega_{y}\right) & \frac{T_{s}}{2}\left({\bar{\omega }}_{z}-\Omega_{z}\right) & \frac{T_{s}}{2}q_{1} & \frac{T_{s}}{2}q_{2} & \frac{T_{s}}{2}q_{3}\\ \frac{T_{s}}{2}\left(\Omega_{x}-{\bar{\omega }}_{x}\right) & 1 & \frac{T_{s}}{2}\left(\Omega_{z}-{\bar{\omega }}_{z}\right) & \frac{T_{s}}{2}\left({\bar{\omega }}_{y}-\Omega_{y}\right) & -\frac{T_{s}}{2}q_{0} & \frac{T_{s}}{2}q_{3} & -\frac{T_{s}}{2}q_{2}\\ \frac{T_{s}}{2}\left(\Omega_{y}-{\bar{\omega }}_{y}\right) & \frac{T_{s}}{2}\left({\bar{\omega }}_{z}-\Omega_{z}\right) & 1 & \frac{T_{s}}{2}\left(\Omega_{x}-{\bar{\omega }}_{x}\right) & -\frac{T_{s}}{2}q_{3} & -\frac{T_{s}}{2}q_{0} & \frac{T_{s}}{2}q_{1}\\ \frac{T_{s}}{2}\left(\Omega_{z}-{\bar{\omega }}_{z}\right) & \frac{T_{s}}{2}\left(\Omega_{y}-{\bar{\omega }}_{y}\right) & \frac{T_{s}}{2}\left({\bar{\omega }}_{x}-\Omega_{x}\right) & 1 & \frac{T_{s}}{2}q_{2} & -\frac{T_{s}}{2}q_{1} & -\frac{T_{s}}{2}q_{0}\\ 0 & 0 & 0 & 0 & 1 & 0 & 0\\ 0 & 0 & 0 & 0 & 0 & 1 & 0\\ 0 & 0 & 0 & 0 & 0 & 0 & 1 \end{array}\right] \end{array}.$$

The measurement update equations utilize the both the observation vector, $z_{k}$ (accelerometer and magnetometer-based attitude realization), and the measurement noise covariance, *R*, to correct the a priori state estimate. First, the Kalman gain matrix $G_{k}$ is obtained, then the state estimate ${\hat{x}}_{k}$ and its error covariance $P_{k}$ are corrected. The a posteriori estimation results are obtained in the following steps.

(22) $$\begin{array}{cc} G_{k} & =P_{k}^{-}H^{T}{\left(HP_{k}^{-}H^{T}+R\right)}^{-1},\\ {\hat{x}}_{k} & ={\hat{x}}_{k}^{-}+G_{k}\left(z_{k}-H{\hat{x}}_{k}^{-}\right),\\ P_{k} & =\left(I-G_{k}H\right)P_{k}^{-}. \end{array}$$

The estimation performance of EKF is mostly determined by the noise covariance matrices *Q* and *R*. Unfortunatelly, in practice, these parameters (i.e., the statistical description of the state and observation noises) are not fully measurable (or require time consuming, complex, and extensive verification and validation procedures); especially in the case of MARG sensors, as the effects of both different noise sources and disturbances are represented with general noise vectors $v_{k}$ and $w_{k}$ in Equations (16) and (17). Generally, the parameters *Q* and *R* are tuned based on engineering intuition through trial-and-error analysis; however, that method yields only a compromise solution between the estimation accuracy and filter dynamics. To overcome this compromise solution, numerical optimization-based approaches have been proposed in our earlier work. The proposed method both allows for evaluation of the best possible (achievable) estimation quality and provides the optimized parameters which maximize the filter performance [1]. We recall this approach to find the optimized parameters of EKF in Section 5.

## 4. Fuzzy-Adaptive Strategy

The adaptive approach varies the noise variances, according to both the instantaneous dynamical behavior and external distrubances, thus providing filter performance superior to that provided by the standard EKF. The instaneous dynamics are characterized by the magnitudes of vibration and external acceleration of the sensor frame. Moreover, the adaptive strategy incorporates the magnitude of the distorted geomagnetic field as an external disturbance. The following subsections present the structure of the adaptive strategy, in which both novel measurement methods of external disturbances and the novel sophisticated fuzzy logic-based inference machine are implemented for the real-time tuning of the noise covariances.

The measurement methods in Section 4.1 and Section 4.2 have been described in detail, with multiple examples and figures, in [1].

### 4.1. Measuring Vibration Magnitude

The system vibration magnitude is described by the oscillation frequency of the sensor frame. For estimation of the instantaneous oscillation frequency, gyroscope readings are utilized, as the sensors provides reliable angular rate measurements for both static and highly dynamic motions. The oscillation frequency is obtained by fast Fourier transform-based (FFT-based) evaluation of short angular rate measurement packets. Let *L* denote the length of these packets. Then, an oscillation frequency estimation $\hat{f}$ is calculated, in three steps, as follows.

1. Collect a measurement packet *x* from the angular rate readings.
2. Obtain frequency domain information about the instantaneous vibration by calculating the discrete Fourier transform of *x*. Let $\left(f_{i},{\left|\Omega \right|}_{i}\right)$ denote the output of FFT, where $f_{i}$ and ${\left|\Omega \right|}_{i}$ represent the frequency components and amplitudes, respectively. The transform of $L_{\mathrm{FFT}}$ length is calculated as
  (23) $$W_{l}=\sum_{k=0}^{L_{\mathrm{FFT}}-1}x_{k}e^{-j\left(\frac{2\pi lk}{L_{\mathrm{FFT}}}\right)},\;l=0,\ldots ,L_{\mathrm{FFT}}-1,$$
  and the output is given by
  (24) $$\left(f_{i},{\left|\Omega \right|}_{i}\right)=\left(\frac{f_{s}i}{L_{\mathrm{FFT}}},\frac{2}{L}\left|W_{i}\right|\right),\;i=0,\ldots ,\frac{L_{\mathrm{FFT}}}{2}.$$
3. Obtain the oscillation frequency estimate $\hat{f}$ by finding the highest-intensity frequency component, such that
  (25) $$\begin{array}{cc} f_{\max} & :\left(f_{\max},{\left|\Omega \right|}_{\max}\right)\wedge {\left|\Omega \right|}_{\max}=\max_{\forall i,f_{i}\leq f_{\mathrm{thr}}}{\left|\Omega \right|}_{i},\\ \hat{f} & =\left\{\begin{array}{cc} 0, & \mathrm{if}\;{\left|\Omega \right|}_{\max}<{\left|\Omega \right|}_{\mathrm{thr}}\\ f_{\max}, & \mathrm{otherwise}\; \end{array}\right., \end{array}$$
  where $f_{\mathrm{thr}}$ denotes the maximum oscillation frequency the system is expected to be exposed to, while the threshold rate magnitude ${\left|\Omega \right|}_{\mathrm{thr}}$ cuts off the noise in the aforementioned evaluation.

### 4.2. Measuring External Acceleration and Magnetic Perturbation Magnitudes

The external acceleration magnitude is calculated based on the accelerometer measurements. The system stays in stationary states (non-accelerating mode) if the magnitude of accelerometer readings is approximately equal to the norm of the reference vector $∥{}_{}^{E}g∥$. Therefore, the external acceleration magnitude $\Delta \alpha_{k}$ can be calculated as the difference between the norms of ${}_{}^{S}A_{k}$ and ${}_{}^{E}g$ in each sampling epoch. As the instantaneous difference does not provide an overall picture of the system dynamics, an accumulated measure is thus utilized to describe the external acceleration magnitude. The accumulated measure ${\hat{\alpha }}_{\mathrm{ext}}$ is formulated as the integrated scalar external acceleration for a window of length *L* (see Equation (26)). This average external acceleration measure provides both useful and broad information of the instantaneous system dynamics.

(26) $${\hat{\alpha }}_{\mathrm{ext}}=\frac{1}{L}\sum_{k=1}^{L}\left|\Delta \alpha_{k}\right|,\;\Delta \alpha_{k}=∥{}_{}^{S}A_{k}∥-∥{}_{}^{E}g∥.$$

The magnetic perturbation magnitude is characterized based on the evaluation of the difference between the norms of ${}_{}^{S}H_{k}$ (instantaneous magnetometer measurement at epoch *k*) and ${}_{}^{E}h$ (reference magnetic field). If no magnetic disturbance is present, then the magnitude of magnetometer measurement is approximately equal to the norm of the reference vector. Otherwise, the magnitude of their difference gives an instantaneous measure of the perturbation magnitude. As it is difficult to draw conclusions based on this brief and instantaneous result at each epoch, similarity to the accelerometer readings, an accumulated measure, is thus applied to quantify the magnetic perturbation magnitude ${\hat{h}}_{\mathrm{ext}}$.

(27) $${\hat{h}}_{\mathrm{ext}}=\frac{1}{L}\sum_{k=1}^{L}\left|\Delta h_{k}\right|,\;\Delta h_{k}=∥{}_{}^{S}H_{k}∥-∥{}_{}^{E}h∥.$$

Similarly to accelerometer and gyroscope sensors, the magnetic perturbation magnitude is determined by collecting data packets of length *L* from the magnetometer and computing the average magnetic field difference using Equation (27).

### 4.3. Fuzzy Inference Machine

The measures $\hat{f}$, ${\hat{\alpha }}_{\mathrm{ext}}$, and ${\hat{h}}_{\mathrm{ext}}$ fully characterize both the instantaneous system dynamics and disturbance magnitudes. These results can be utilized in an inference system in which the noise covariance manipulation of the EKF is described according to the external effects. As a result, an adaptive strategy is established that (online) tunes the noise covariances as a function of the measures $\hat{f}$, ${\hat{\alpha }}_{\mathrm{ext}}$, and ${\hat{h}}_{\mathrm{ext}}$.

The relationships between the aforementioned measures and the EKF parameters are defined with fuzzy reasoning. Fuzzy logic does not require complex mathematical models from the system designer but, instead, it enables the implementation of deductions easily and effectively by using fuzzy sets and simple IF-THEN linguistic rules. Therefore, heuristic knowledge and a collection of deductions make such an inference system realizable. The fuzzy inference system is executed in three main steps: fuzzification determines the membership values of the crisp input variables, inference machine translates the heuristic knowledge and assigns a firing value to each fuzzy output, and defuzzification maps the fuzzy output to the crisp domain. The main parts of the algorithm are detailed in [67].

Observations related to the system behavior and human common-sense contribute to collecting the empirical IF-THEN rules (deductions) that define the fuzzy inference machine. In the case of attitude estimation with MARG sensors, the main deductions are as follows.

- IF the sensor frame stays in stationary (non-accelerating and non-perturbed) mode, THEN a well-chosen ratio between the noise covariances *Q* and *R* yields satisfactory state estimation performance.
- As the external disturbance effects are absorbed by the measurement noise $v_{k}$ in Equation (17), IF vibration, external acceleration, and magnetic perturbations disturb the MARG-based attitude realization, THEN the measurement noise covariance *R* should be increased according to the intensity of the measures $\hat{f}$, ${\hat{\alpha }}_{\mathrm{ext}}$, and ${\hat{h}}_{\mathrm{ext}}$ (i.e., higher noise variance characterizes the attitude realization $q_{k,\mathrm{TRIAD}}$ with higher uncertainty).

**Figure 2:**
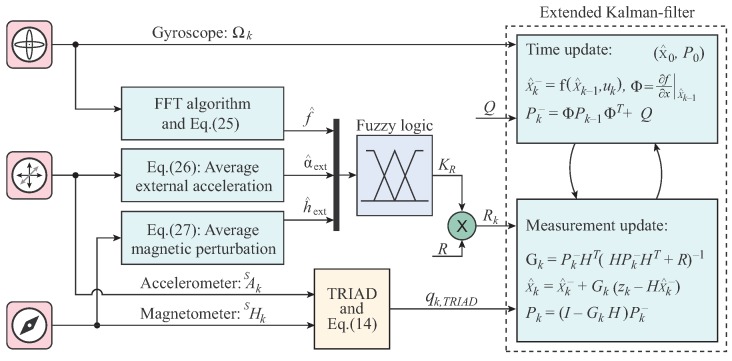
Structure of the FAEKF.

**Figure 3:**
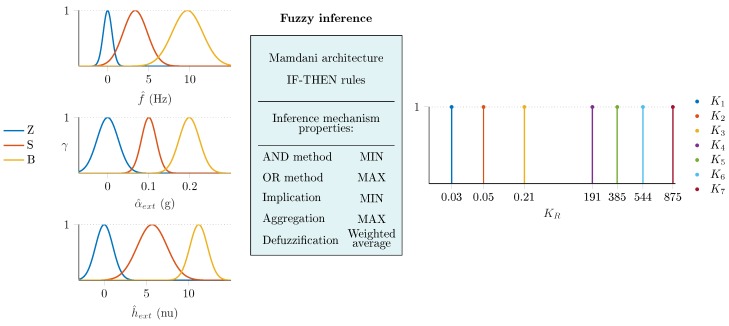
Properties of the applied fuzzy inference machine.

**Figure 4:**
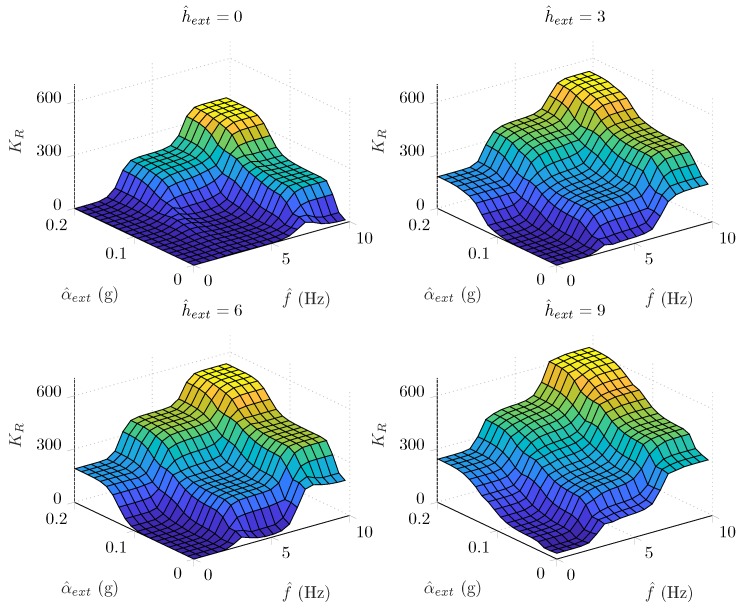
Generated surfaces related to the fuzzy rule base.

The overall FAEKF structure is depicted in Figure 2, where a three-input one-output fuzzy inference machine executes the online tuning of noise variances. The inputs of the fuzzy system are the measures $\hat{f}$, ${\hat{\alpha }}_{\mathrm{ext}}$, and ${\hat{h}}_{\mathrm{ext}}$, whereas weighting factors, denoted by $K_{R}$, are output weights for the *R* parameter (i.e., the adaptive strategy varies the measurement noise covariance matrix in each epoch *k* as $R_{k}=K_{R,k}R$). The ranges of the input variables $\hat{f}\;\left(\mathrm{Hz}\right)$, ${\hat{\alpha }}_{\mathrm{ext}}\;\left(g\right)$, and ${\hat{h}}_{\mathrm{ext}}\;(\mathrm{normalized}\;\mathrm{unit},\;\mathrm{nu})$, as well as the output variable $K_{R}$, were selected based on our earlier research results in [1]. Three Gaussian membership functions cover each input range, where the magnitudes of $\hat{f}$, ${\hat{\alpha }}_{\mathrm{ext}}$ and ${\hat{h}}_{\mathrm{ext}}$ are characterized by Z (zero), S (small), and B (big) fuzzy sets. The output ranges were covered with seven singleton consequents ($K_{1},\cdots ,K_{7}$), which represent the scaling magnitudes. Both the applied membership functions and fuzzy inference system properties are depicted in Figure 3. The fuzzy surfaces expressing the relationships between the crisp inputs and outputs are depicted in Figure 4.

A sophisticated inference system was implemented, where the initial deductions described above were expanded into 27 rules. These simple IF-THEN linguistic rules completely describe the scaling of noise variances, according to the magnitudes of the external acceleration, vibration, and magnetic perturbation. The implemented rule base for $K_{R}$ is summarized in Table 1. Two examples describe the interpretation of the implemented inference system, as follows:

1. IF the oscillation frequency $\hat{f}$ is zero (Z) and the external acceleration ${\hat{\alpha }}_{\mathrm{ext}}$ and magnetic perturbation ${\hat{h}}_{\mathrm{ext}}$ magnitudes are big (B), THEN a fairly large scaling factor ($K_{R}=K_{5}$) is applied for the measurement noise covariance. This collocation of the system state means that the observation is expected to have rather large uncertainty and, therefore, the algorithm relies more heavily on the state propagation (left side, second row, second column).
2. IF $\hat{f}$ is small (S) and the ${\hat{\alpha }}_{\mathrm{ext}}$ and ${\hat{h}}_{\mathrm{ext}}$ measures are close to zero (Z), THEN a smaller weight of $K_{R}=K_{2}$ is applied for *R*. Therefore, the algorithm considers the observation with higher reliability and maintains the correction of the state propagation by processing the measurements with higher significance (middle, first row, first column).

The crisp scaling factor is computed by weighted average-based defuzzification of the fuzzy output, in three steps:

1. Fuzzification of the observation vector $\chi =\left({\hat{f}}_{k},{\hat{\alpha }}_{ext,k},{\hat{h}}_{ext,k}\right)\in \hat{f}\times {\hat{\alpha }}_{ext}\times {\hat{h}}_{ext}$ and calculation of the firing values of the ith rule (i=1,…,27). Let $\chi_{j}$ denote the jth dimension of the observation vector (j=1,2,3), then the firing value $\gamma_{j}^{i}$ represents the fitting degree of the observation $\chi_{j}$ to the antecedent fuzzy set $X_{j}^{i}$ in the jth dimension of the ith rule as
  (28) $$\begin{array}{c} \gamma_{j}^{i}=\max_{\chi_{j}}\left\{\min\left\{X_{j}^{*}\left(\chi_{j}\right),X_{j}^{i}\left(\chi_{j}\right)\right\}\right\},\;X_{j}^{*}\left(\chi_{j}\right)=e^{-\frac{{\left(\chi_{j}-b_{ij}\right)}^{2}}{2c_{ij}^{2}}}, \end{array}$$
  where $X_{j}^{*}\left(\chi_{j}\right)$ is the fuzzified observation, moreover, $b_{ij}$ and $c_{ij}$ denote the mean and standard deviation of the Gaussian function antecedent defined in the $j^{\mathrm{th}}$ dimension of the $i^{\mathrm{th}}$ rule.
2. Calculation of the applicability measure of the $i^{\mathrm{th}}$ rule, denoted by $\gamma^{i}$, as the minimum of the aforementioned firing values. This weight determines the significance of the consequent fuzzy set $\kappa^{i}$ defined in the $i^{\mathrm{th}}$ rule.
  (29) $$\begin{array}{c} \gamma^{i}=\min_{j=1}^{3}\gamma_{j}^{i} \end{array}$$
3. Computation of the crisp output *K* as the weighted average over all rule outputs:
  (30) $$\begin{array}{c} K=\frac{\sum_{i=1}^{27}\kappa^{i}\;\cdot \;\gamma^{i}}{\sum_{i=1}^{27}\gamma^{i}}. \end{array}$$

The proposed fuzzy inference machine is a zero-order Sugeno system. The complete inference for the adaptive measurement noise covariances in each epoch *k* can be given in a compact form as

(31) $$\begin{array}{c} K\left(\kappa ,\hat{f},{\hat{\alpha }}_{\mathrm{ext}},{\hat{h}}_{\mathrm{ext}}\right)=\frac{\sum_{i=1}^{27}\kappa^{i}\;\cdot \;\min\left(\gamma^{i}\left(\hat{f}\right),\min\left(\gamma^{i}\left({\hat{\alpha }}_{\mathrm{ext}}\right),\gamma^{i}\left({\hat{h}}_{\mathrm{ext}}\right)\right)\right)}{\sum_{i=1}^{27}\min\left(\gamma^{i}\left(\hat{f}\right),\min\left(\gamma^{i}\left({\hat{\alpha }}_{\mathrm{ext}}\right),\gamma^{i}\left({\hat{h}}_{\mathrm{ext}}\right)\right)\right)},\\ R_{k}=K_{R,k}R,\;K_{R,k}=K\left(\kappa_{R},{\hat{f}}_{k},{\hat{\alpha }}_{ext,k},{\hat{h}}_{ext,k}\right),\;\kappa_{R}={\left(K_{1},\ldots ,K_{7}\right)}^{T}, \end{array}$$

where $\gamma^{i}\left(\hat{f}\right)$, $\gamma^{i}\left({\hat{\alpha }}_{\mathrm{ext}}\right)$, and $\gamma^{i}\left({\hat{h}}_{\mathrm{ext}}\right)$ are the $i^{\mathrm{th}}$-rule fired membership function values and $\kappa^{i}$ denotes the singleton value of the consequent weighting factor of the $i^{\mathrm{th}}$ rule for scaling the noise covariance *R* (see Figure 2 and Figure 3).

## 5. Experimental Validation

This section describes the test platform employed in the evaluation of filter performance, the optimization approach utilized to tune the filter parameters, and the attitude determination results during different dynamic motions and external perturbations.

### 5.1. Test Environment

A comprehensive framework was designed, in which a 6 DOF test bench dynamically altered the pose (position and orientation) of a MARG unit. The 6 DOF test bench was utilized to both simulate various (accelerating, non-accelerating, and vibrating) dynamic behaviors and measure the real attitude of the sensor frame, along with the raw MARG data. The framework was based on the widely used ROS and the Gazebo open source dynamics simulator, which utilizes physics engines to consider the effects of gravity, friction, and forces [68]. As a result, this framework enabled the evaluation of state estimation error, quantification of the filter performance, and tuning of filter parameters.

**Figure 5:**
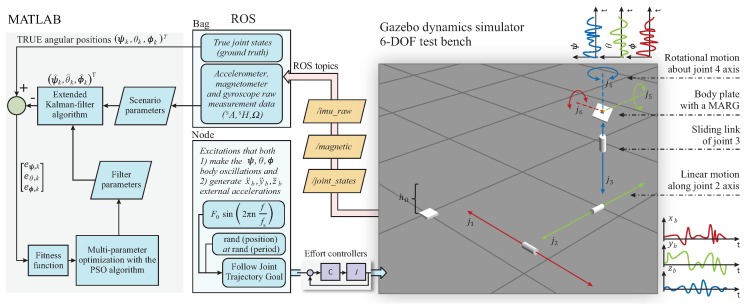
Block diagram of the test environment and filter tuning procedure (video of the closed-loop in [58]).

The proposed test bench consisted of three prismatic joints and three revolute joints. The prismatic joints made the sensor frame slide back and forth, up and down in the three dimensional (3D) space by three 3m long rails. The revolute joints set the instantaneous attitude (Euler angles) of the sensor frame. The MARG unit is attached to a plate at the end of this kinematic chain and, so, the 6 DOF system enabled both the spatial coordinates and orientation of the sensor frame to be set and measured. Moreover, this 6 DOF mechanism enabled the generation of external accelerations simultaneously with sensor frame oscillations. Therefore, a variety of dynamic (vibrating and accelerating) system conditions could be simulated, where both the raw sensor data and real joint states were be recorded. Figure 5 shows the model of the test environment in Gazebo.

Let $x_{b}$, $y_{b}$, and $z_{b}$ denote the spatial coordinates of the body plate (i.e., the origin of the MARG unit). Then, the total kinetic energy *T* of the test platform is given as

(32) $$\begin{array}{cc} T & =\frac{1}{2}{\dot{q}}^{T}M_{\mathrm{mass}}\dot{q},\;M_{\mathrm{mass}}=\mathrm{diag}\left(\left(m_{j}+I_{3\times 1}m_{b},J_{b}\right)\right), \end{array}$$

where $m_{j}={\left(m_{j,1},m_{j,2},m_{j,3}\right)}^{T}$, $m_{j,i}$ denotes the mass of each prismatic joint for i={1,2,3}, whereas $m_{b}$ and $J_{b}={\left(J_{b,\phi },J_{b,\theta },J_{b,\psi }\right)}^{T}$ indicate the mass and moment of inertia of the body plate, respectively. Moreover, $q={\left(x_{b},y_{b},z_{b},\phi ,\theta ,\psi \right)}^{T}$ denotes the vector of generalized coordinates. The potential energy stored in the system is approximated as $P=\left(m_{b}+\sum_{i}^{3}m_{j,i}\right)gh_{0}+\left(m_{b}+m_{j,3}\right)gz_{b}-m_{j,3}gh_{1}$, where the constants $h_{0}$ and $h_{1}$ denote the base height and distance between the body plate and third prismatic joint, respectively. The Lagrange function of the system is L=T−P, where the motion of the system can be determined with the help of the Lagrange equations [69]. As a result, the equations of motion can be written in the following form,

(33) $$M\left(q\right)\ddot{q}+V\left(q,\dot{q}\right)=\tau_{a}-\tau_{f},$$

where $M\left(q\right)$ is the inertia matrix, $V\left(q,\dot{q}\right)$, including the Coriolis, centrifugal, and potential force terms, whereas $\tau_{a}$ and $\tau_{f}$ indicate the generalized external torques and friction effects, respectively.

The aforementioned dynamics were implemented in an Unified Robot Description Format (URDF) file [70]. This file enables the specification of the whole geometric description of the system, including the robot kinematics, motion ranges, location of frames, mass properties, and collisions. Each joint (DOF) is driven in a closed-loop with an independent proportional-integral-derivative (PID) effort controller. Each effort controller was implemented, using the *ros controllers* meta-package, as a single-input single-output (SISO) low-level controller, in which torque control action was applied to the joint. The PID parameters were set up heuristically by iterative tuning in Gazebo. The true linear and angular positions of each joint were supplied by the *joint state controller*, a sensor controller that publishes the joint state information (i.e., true positions, velocities, and efforts are represented in double-precision floating-point format without measurement noise, discrepancy, or delay) [71,72]. In this application, the joint state information was obtained with a $f_{s}=1\;\mathrm{kHz}$ sampling frequency. The sensor measurements were provided by independent Gazebo plugins, developed in [73]. These IMU and magnetic field sensor plugins were attached to the body plate of the 6 DOF test bench by including them in the URDF file.

To execute different acceleration and vibration dynamic motions, on one hand, random desired values were generated with random frequencies for the PID controllers of the three prismatic joints in their configuration space. On the other hand, different sinusoidal signals were supplied as reference values to the PID controllers of the three revolute joints, where both the amplitude and frequency were varied randomly. Therefore, the closed-loop system caused the 6 DOF mechanism to execute a wide variety of dynamic movements in the 3D space, with continously varying oscillations and accelerations. Simultaneously, the three prismatic joints made the sensor frame slidd back and forth, as well as up and down; simulating various external accelerations. The true joint states, along with the instantaneous MARG sensor data, were collected to evaulate the attitude estimation performance.

Video demonstrations of the closed-loop dynamics have been shared on our website. Moreover, as was described in Section 1, the whole ROS package, which includes the test bench properties, URDF files, applied effort controllers, and Gazebo configuration files, have been made publicly available in the supplementary online material, to help other lab teams evaluate similar experiments [58].

#### Magnetic Perturbations

Magnetic perturbations were generated artificially, as the Gazebo simulation environment does not contain such a feature. Therefore, based on the experimental results with magnetic disturbances conducted in [74,75,76,77], we developed a simple algorithm to generate magnetic perturbations. The algorithm is composed of three main steps, which are described as follows.

1. Generate a perfect artificial signal *m* of length $L_{m}$ as a mixture of square, saw-tooth, triangle, and two sinusoidal signals. Both the sequence of these signals and their parameters (i.e., the amplitude and frequency) are randomly selected.
2. Obtain the analytic signal $m_{a}$ from *m*, where the real part is the original signal, while the imaginary part contains the Hilbert transform (i.e., the original signal with a π/2 phase shift [78]). Then, generate the artificial perturbation $m_{p}$ as the sum of the imaginary part and absolute value of the Hilbert transformed complex signal, where the sequence of absolute values is reversed in time:
  (34) $$\begin{array}{cc} m_{a,k} & =m_{r,k}+jm_{i,k},\;k=1,\ldots ,L_{m},\\ m_{p,k} & =m_{i,k}+\left|m_{a,l}\right|,\;k=1,\ldots ,L_{m},\;l=L_{m},\ldots ,1. \end{array}$$
3. Remove the continuous linear trend of $m_{p}$ and low-pass filter the detrended signal with a first order Butterworth infinite impulse response (IIR) filter.

**Figure 6:**
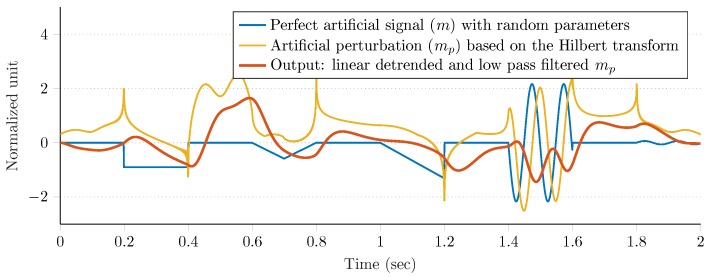
Demonstration of the proposed magnetic perturbation generator algorithm.

**Figure 7:**
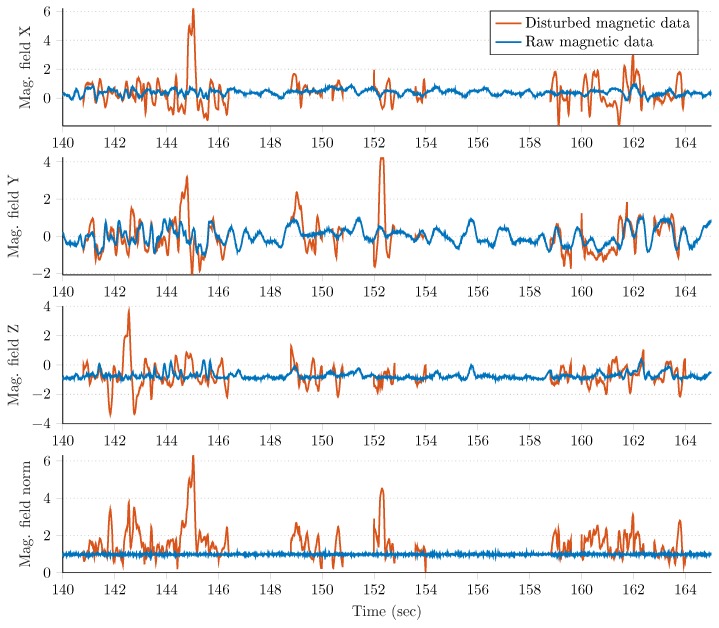
Magnetic field measurements before and after the application of the magnetic perturbation generator algorithm.

Each step of the aforementioned algorithm is depicted in Figure 6. Moreover, Figure 7 highlights the effect of the artificial perturbation on both the norm and each component of the raw magnetometer signal. The blue curves represent the raw (calibrated, undisturbed, and normalized) magnetometer measurements and the red curves show random sections, where the magnetometer had been disturbed artificially with the proposed algorithm. These figures illustrate that the algorithm enabled both generation of realistic magnetic perturbation effects and incorporation of effects of this type of disturbance into the analysis of attitude estimation.

### 5.2. Tuning of Filter Parameters

Tuning of the filter parameters was executed in MATLAB on a training data set collected in the aforementioned test environment. The heuristic particle swarm optimization (PSO) algorithm was utilized for the filter tuning problem, as it does not require gradient information, guides the search well even in nonlinear noisy systems, and has demonstrated greater effectiveness and robustness than other optimization methods [79,80,81]. Both the algorithm and applied PSO-based optimization procedure have been presented in detail in our earlier works [1,82,83]; therefore, only key information is described in the following paragraphs.

The inputs of the optimization problem are the real angular positions (i.e., the true Euler angles $\phi_{k}$, $\theta_{k}$, and $\psi_{k}$ provided by the 6 DOF test bench) and MARG sensor data (i.e., the acceleration, angular velocity, and magnetic field measurements), whereas its outputs are the estimation errors $e_{\phi ,k}=\phi_{k}-{\hat{\phi }}_{k}$, $e_{\theta ,k}=\theta_{k}-{\hat{\theta }}_{k}$, and $e_{\psi ,k}=\psi_{k}-{\hat{\psi }}_{k}$, where ${\hat{\phi }}_{k}$, ${\hat{\theta }}_{k}$, and ${\hat{\phi }}_{k}$ denote the estimated Euler angles (i.e., the outputs of the implemented filter algorithm). The PSO is a population-based search algorithm that guides the search in the search space by employing a fitness function. In our work, a complex fitness function was formulated for the problem to both quantify the differences between the true and estimated Euler angles and measure the overall filter performance. Three mean squared errors (MSE) were combined to evaluate the filtration quality. The PSO-based minimization of the following fitness function enabled the filter parameters to be successfully tuned:

(35) $$\begin{array}{c} F=\sqrt[{3}]{\prod_{j=1}^{k_{\mathrm{sce}}}\left(\frac{\sum_{k=1}^{N_{j}}e_{\phi ,k}^{2}}{N_{j}}\right)\left(\frac{\sum_{k=1}^{N_{j}}e_{\theta ,k}^{2}}{N_{j}}\right)\left(\frac{\sum_{k=1}^{N_{j}}e_{\psi ,k}^{2}}{N_{j}}\right)}, \end{array}$$

where $k_{\mathrm{sce}}$ denotes the number of scenarios taken into account in the optimization problem; $N_{j}$ is the measurement length in the $k_{\mathrm{sce}}^{\mathrm{th}}$ scenario; and $e_{\phi ,k}$, $e_{\theta ,k}$, and $e_{\psi ,k}$ indicate the roll, pitch, and yaw estimation errors, respectively. The optimization algorithm determined the optimal possible filter parameters, corresponding to the lowest possible fitness function value. The block diagram of the filter parameter optimization procedure is depicted in Figure 5.

The optimization could begin running once the parameters were initialized. The PSO parameters were selected based on earlier studies [84,85], whereas the filter parameters (${\hat{x}}_{0}$, $P_{0}$, *Q*, and *R*) were initialized by employing the results presented in [1]. As the sampling time in the ROS-based framework was relatively low ($T_{s}=1\mathrm{ms}$), the adaptive strategy could be executed with bigger window size of L=400; moreover, the length of the transform was $L_{\mathrm{FFT}}=2^{9}$ and the threshold oscillation frequency and amplitude were $f_{\mathrm{thr}}=10\;\mathrm{Hz}$ and ${\left|\Omega \right|}_{\mathrm{thr}}=0.26\;\mathrm{rad}/s$, respectively. The process noises $\mu_{k}^{q}$ and $\nu_{k}$ in Equation (16) were considered to be statistically independent [1,19,41]; therefore, diagonal matrices were applied for both the process and measurement noise covariances with the following characteristics,

(36) $$\begin{array}{c} Q=\left[\begin{array}{cc} I_{4}\;\cdot \;Q_{q} & 0_{3\times 4}\\ 0_{4\times 3} & I_{3}\;\cdot \;Q_{\bar{\omega }} \end{array}\right],\;R=I_{4}\;\cdot \;\rho , \end{array}$$

where the $Q_{q}$, $Q_{\bar{\omega }}$, and ρ constant noise variances were tuned with PSO. Namely, the optimization converged the quaternion measurement noise variance to a higher value of ρ=3.53. This outcome was expected, as intense accelerations and vibrations were applied with the 6 DOF test bench and the effects of these external distrubances were absorbed in the measurement noise $v_{k}$ in Equation (17). This high-noise variance value indicates that the TRIAD-based attitude realization was significantly more unreliable than the gyro-based state propagation, especially in highly dynamic states of the system. At the same time, the process noise variances converged to noticeably small values (i.e., $Q_{q}=1.45\times 10^{-6}$ and $Q_{\bar{\omega }}=9.71\times 10^{-10}$), resulting in the state-space dynamics (Equation (16)) becoming much more reliable than the measurement correction equations. The successful optimization contributed to finding the tuned EKF parameters which provided satisfactory attitude estimation quality with the help of the adaptive strategy described in Section 4 for both static and extreme (vibrating and accelerating) dynamic conditions.

### 5.3. Results

The attitude estimation performance of the FAEKF was evaluated on three measurements performed in the test environment (Measurements 1–3 lasted for approximately 160s, 210s, and 315s, respectively). The dynamic motions executed by the 6 DOF test bench during these measurements included stationary states, slow and fast changes in angular positions, mild and intense oscillations, and external accelerations. The dynamic circumstances in which the filter performance was investigated were characterized by the following ranges; 0−8Hz for sensor frame oscillation frequency, ±50rad/s for angular velocity, ±16g for external spatial acceleration, and 0−5nu for magnetic perturbation magnitude.

**Figure 8:**
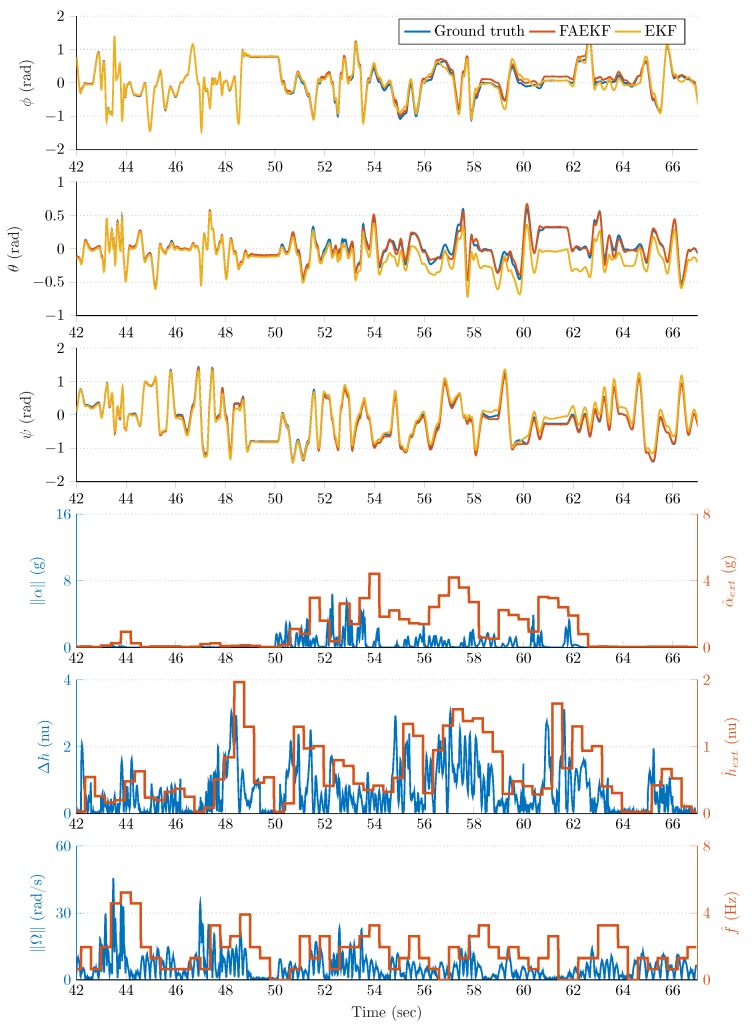
First time slot from the measurements.

**Figure 9:**
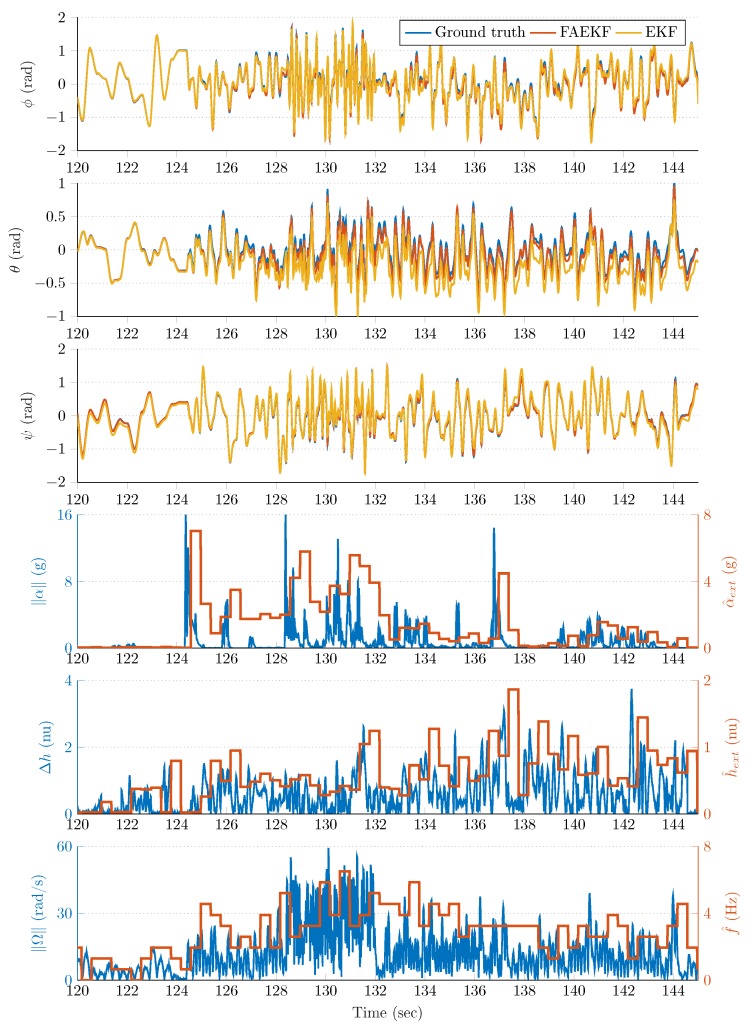
Second time slot from the measurements.

**Figure 10:**
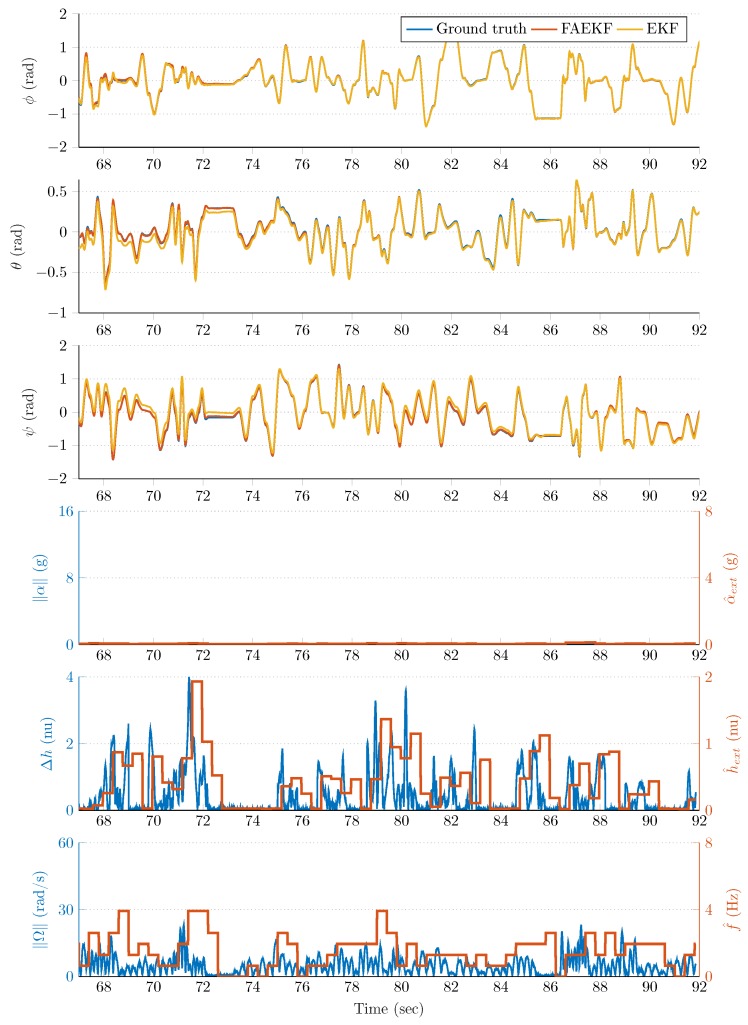
Third time slot from the measurements.

**Figure 11:**
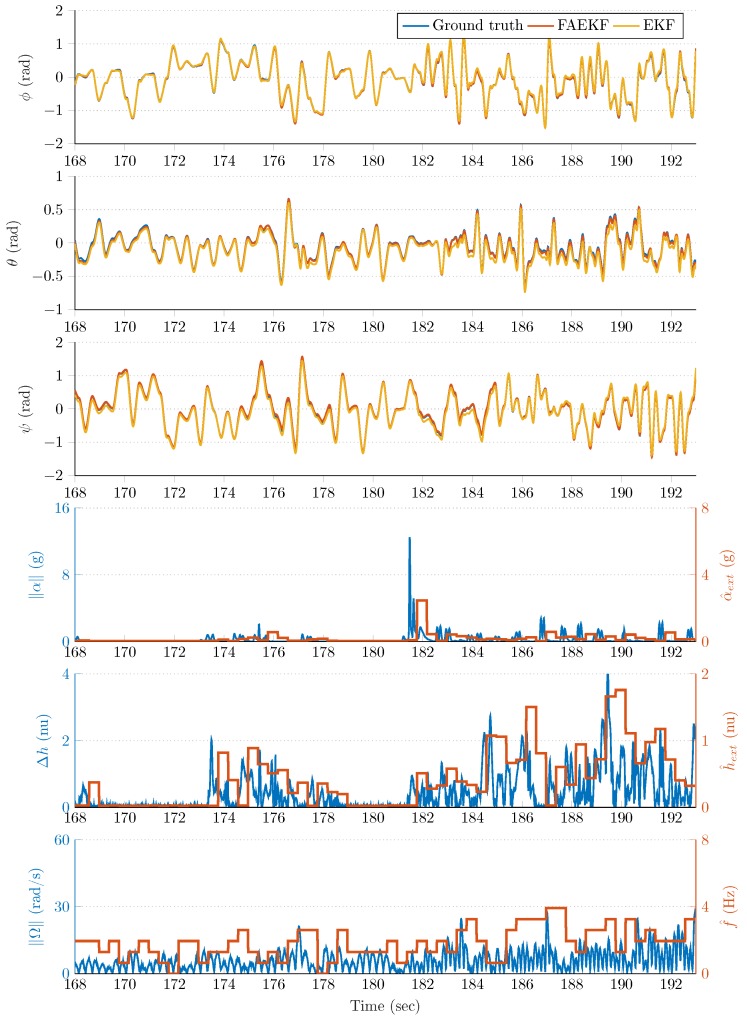
Fourth time slot from the measurements.

The robust filter performance in the highly disturbed (accelerating and vibrating) test environment is highlighted in Figure 8, Figure 9, Figure 10 and Figure 11. The first three rows of each figure show the roll (ϕ), pitch (θ), and yaw (ψ) angles, where the blue curve indicates the true Euler angles (obtained by the joint states ROS topic), whereas the red and yellow curves highlight the attitude estimation with and without the proposed fuzzy adaptive strategy, respectively. The fourth rows depict both the instantaneous external acceleration (blue curves) executed by the 6 DOF test bench and average dynamic acceleration (red curves) determined by Equation (26). Similarly, the blue curve of the fifth row of each figure shows the instantaneous magnetic perturbation generated by Equation (34), whereas the red curve indicates the average magnetic field difference calculated by Equation (27). Finally, the sixth row depicts both the instantaneous angular rate magnitude (blue curves) and the oscillation frequency of the sensor frame (red curves) determined by Equation (25). The last three rows illustrate that the employed measurement methods in the adaptive strategy provided useful information related to the external acceleration, vibration, and magnetic disturbance magnitudes.

The noticable performance improvement provided by the fuzzy-adaptive strategy is highlighted both by the figures and the results included in Table 2. The curves corresponding to the FAEKF output fit to the true Euler angles to a satisfactory degree, both in frequencies and amplitudes; even when extreme external perturbations were present. The effects of these disturbances drastically decreased the performance of the standard EKF. For example, Figure 8 highlights that, at approximately 50s, an increased external acceleration and magnetic perturbation influenced the attitude estimation over a 15s long period. During this period, the effects of these disturbances were effectively suppressed by the FAEKF; the adaptation laws enabled it to achieve satisfactory filter accuracy and convergence. It is also shown that, without the fuzzy-adaptive strategy, an unsatisfactory EKF performance was provided (Euler angles indicated with yellow curves). A similar outcome can be observed in Figure 9; namely, the external acceleration and magnetic perturbation effects in the high vibrating environment contributed to a significant decrease in the EKF estimation quality (e.g., see the yellow curves at ~140s). However, it is also shown that the adaptation laws enabled cancellation of these effects, even under diverse dynamic conditions. Under static conditions, both EKF and FAEKF provided approximately the same performance levels; these results are highlighted in Figure 10 and Figure 11. As low-frequency oscillations along with no magnetic perturbation nor external acceleration enabled the accelerometer- and magnetometer-based attitude realization (TRIAD output) to be characterized with high accuracy, the EKF could therefore provide satisfactory estimation quality based on the implemented state-space model. In these static cases, the adaptive strategy does not modify the noise variances, as the well-chosen ratio between the covariance parameters yields a satisfactory estimation quality.

The filter performance was quantified with the mean squared error (MSE) and standard deviation (STD) of the attitude estimation error. These results were calculated for each measurement (M1, M2, and M3) and are summarized in Table 2. Based on the results, a significant improvement in the overall filter performance can be observed. In each measurement case, the yaw angle estimation was characterized by the smallest errors, while slightly less robust outputs were provided for the roll and pitch angle estimation. This outcome was expected in our configuration and is related to the TRIAD algorithm’s characteristics. Namely, the impact of magnetometer readings relative to the vertical axis is eliminated in ${\hat{s}}_{2}$ and ${\hat{r}}_{2}$ (see Equations (11) and (12)), therefore the pitch and roll angles were determined based on only the accelerometer measurements [23]. As the accelerometer measurements were disturbed much more heavily (via both the measurement noise and frequent external accelerations) than the magnetometer readings, therefore the disturbances influenced slightly more the roll and pitch estimation performance of the filter. Based on both the figures and Table 2, it can be concluded that a superior estimation convergence was achieved with the introduced adaptive strategy, thereby validating the performance of the proposed FAEKF approach. Nevertheless, some potential improvements are left open for investigation in future studies:

1. Employing a more robust deterministic approach to determine the quaternion from accelerometer and magnetometer observations in the measurement update state of the EKF.
2. Partitioning the fuzzy inputs and outputs into additional fuzzy sets, thereby implementing a more advanced fuzzy inference system.
3. Tuning the shapes of the applied fuzzy sets, the ranges of input and output variables, and the weights of the IF-THEN rules with the aid of optimization.
4. Varying the window size in the determination of external disturbance magnitudes, thereby providing more accurate measures for the adaptation laws.
5. Extending the state space model with external acceleration and magnetic perturbation models, where the driving Gaussian variables vary according to the external disturbance magnitudes.
6. Applying an additional output in the fuzzy inference system which weights the process noise covariance matrix.

Throughout the results, it was demonstrated that the developed methods in the adaptive strategy provided relevant information of the environment in which attitude estimation was performed. The obtained external disturbance magnitudes enabled us to form an inference mechanism that effectively manipulated the noise variances on-the-fly, thereby providing superior filter performance. As external accelerations, magnetic perturbations, and vibration are common disturbance sources in motorized mechatronic systems, the proposed method can be advantegously applied in such mechatronic systems. The paper also demonstrated the benefits of fuzzy logic, as it provided an expert-oriented approach to implementing complex relations with the help of simple heuristic IF-THEN rules. The proposed adaptation laws can be universally applied for the online tuning of any filter structure. Moreover, both the measurement methods and fuzzy inference mechanism can be intelligently employed in adaptive control solutions for mechatronic systems performing motions in unknown and/or disturbed environments (e.g., wheeled/legged robots moving on uneven terrain or UAVs maneuvering in windy environments).

## 6. Conclusions

This paper proposed a novel qAEKF structure, FAEKF, in which both new measurement techniques were developed for the calculation of external disturbance magnitudes and novel adaptation laws were implemented with fuzzy logic. The EKF core incorporated a 7-dimensional state-space model for the estimation of both the quaternion and gyroscope bias vector. Three external disturbance measurement methods were fused to characterize the dynamic and/or perturbed environment in which attitude estimation was being performed. Namely, the external acceleration and magnetic disturbance magnitudes were represented with accumulated measures, which provided broad information of the instantaneous system circumstances. Moreover, the instantaneous oscillation frequency of the sensor frame was obtained as a measure of vibration magnitude. A sophisticated fuzzy inference machine was designed, which formed the relationship between these external disturbance magnitudes and the EKF parameters. The implemented fuzzy system incorporated simple heuristic IF-THEN rules, in order to consistently modify the noise variance values of the filter, thus providing accurate and robust attitude results. A novel test environment was designed for experimental validation of the proposed techniques, in which a 6 DOF test bench both executed various dynamic motions and measured the true Euler angles along with the raw MARG data. The tuning of EKF parameters was performed with the aid of the PSO algorithm. The experimental results showed that the proposed adaptive structure effectively suppresses the effects of external disturbances, thereby enabling the FAEKF to provide reliable attitude estimation results, even in extreme dynamic and/or perturbed situations. The proposed adaptive (dynamic-dependent) feature makes the FAEKF a suitable candidate for attitude estimation in mechatronic systems operating in variable conditions. Future work will involve the performance evaluation of the filter during the control of our Szabad(ka)-II hexapod robot in a disturbed environment.

## Figures and Tables

**Table 1:** Rule base of the fuzzy inference machine.

Vibration $\hat{f}=Z$		Mag. pert. ${\hat{h}}_{\mathrm{ext}}$				Vibration $\hat{f}=S$		Mag. pert. ${\hat{h}}_{\mathrm{ext}}$				Vibration $\hat{f}=B$		Mag. pert. ${\hat{h}}_{\mathrm{ext}}$		
		Z	S	B				Z	S	B				Z	S	B
Ext. acc. ${\hat{\alpha }}_{ext}$	Z	$K_{1}$	$K_{2}$	$K_{3}$		Ext. acc. ${\hat{\alpha }}_{ext}$	Z	$K_{2}$	$K_{3}$	$K_{4}$		Ext. acc. ${\hat{\alpha }}_{ext}$	Z	$K_{3}$	$K_{4}$	$K_{5}$
	S	$K_{2}$	$K_{3}$	$K_{4}$			S	$K_{3}$	$K_{4}$	$K_{5}$			S	$K_{4}$	$K_{5}$	$K_{6}$
	B	$K_{3}$	$K_{4}$	$K_{5}$			B	$K_{4}$	$K_{5}$	$K_{6}$			B	$K_{5}$	$K_{6}$	$K_{7}$

**Table 2:** Mean squared error (MSE) and standard deviation (STD) results of the investigated filters.

Condition		roll (ϕ)		pitch (θ)		yaw (ψ)	
		MSE (${\mathrm{rad}}^{2}$)	STD (rad)	MSE (${\mathrm{rad}}^{2}$)	STD (rad)	MSE (${\mathrm{rad}}^{2}$)	STD (rad)
M1	**FAEKF**	0.0010	0.0301	0.0026	0.0421	0.0004	0.0188
	**EKF**	0.0037	0.0605	0.0127	0.0927	0.0099	0.0688
M2	**FAEKF**	0.0020	0.0433	0.0040	0.0536	0.0007	0.0261
	**EKF**	0.0089	0.0937	0.0252	0.1261	0.0085	0.0916
M3	**FAEKF**	0.0050	0.0695	0.0056	0.0548	0.0016	0.0405
	**EKF**	0.0046	0.0669	0.0102	0.0650	0.0089	0.0944
